# Heterogeneous TiO_2_@Nb_2_O_5_ composite as a high-performance anode for lithium-ion batteries

**DOI:** 10.1038/s41598-017-07562-5

**Published:** 2017-08-03

**Authors:** Yubin Liu, Liwei Lin, Weifeng Zhang, Mingdeng Wei

**Affiliations:** 1 State Key Laboratory of Photocatalysis on Energy and Environment, Fuzhou University, Fuzhou, Fujian, 350002 China; 2 Institute of Advanced Energy Materials, Fuzhou University, Fuzhou, Fujian, 350002 China

## Abstract

Heterogeneous TiO_2_@Nb_2_O_5_ composites, in which TiO_2_ nanoparticles were evenly embedded on ultrathin Nb_2_O_5_ nanosheets, were used as anode materials for LIBs and demonstrated high capacities and excellent rate capability. For instance, this material displayed large capacities of 166.3 and 129.1 mA h g^−1^ at current densities of 1 A g^−1^ after 100 cycles and 5 A g^−1^ after 300 cycles, respectively.

## Introduction

To meet the demand for large application of lithium-ion batteries (LIBs) in the field of hybrid electric vehicles and electric vehicles, development of electrode materials with high capacity and superior rate capability is urgent^1–3^. Over the past few decades, TiO_2_ have been considered as an alternative anode material for LIBs because of chemical stability, safety, environmental friendliness as well as a negligible volume change in Li-ion intercalation^4–8^. In particular, nanostructured TiO_2_, with short electron/ion diffusion distance and high surface area, exhibit enhanced lithium-ion intercalation properties^9–12^. Notably, ultrafine TiO_2_ with several nanometers can reveal pseudocapacitance storage process as well as lithium intercalation process, resulting in an increase of the total amount of Li storage and improved rate capability^13, 14^. However, nanostructured TiO_2_ are prone to suffer from server aggregation which dramatically diminishes its rate performances and cycling stabilities.

Dispersion of nanosized TiO_2_ onto other materials with a large surface area is one of the strategies to overcome the above drawback of TiO_2_, because they can efficiently inhibit aggregation of TiO_2_. Conventionally, carbonaceous materials have been considered as excellent supports for TiO_2_ because of its excellent electronic conductivity. Thus, TiO_2_@carbon composite nanofibers^15^, TiO_2_/carbon nanotubes^16^ and TiO_2_-mesoporous carbon nanocomposites^17^ have been explored for lithium storage. Among these carbonaceous materials, the graphene with a two-dimensional structure appears particularly promising to improve the electrochemical performance of TiO_2_ composite materials and served as a support for nanostructured TiO_2_ due to its superior electrical conductivity, large surface area and excellent structural flexibility^18–24^. For examples, Wang *et al*. used anionic sulfate surfactants to synthesize TiO_2_-graphene hybrid, which showed improved electrochemical performance^25^. Zhang *et al*. reported a simple one-step hydrothermal method toward *in situ* growth of mesoporous TiO_2_ on 3D-graphene aerogels, which displayed a reversible capacity of 99 mA h g^−1^ at a high rate of 5000 mA g^−1^ ^26^. Nevertheless, a homogenous dispersion of TiO_2_ nanoparticles onto graphene remains a challenge because of intrinsic incompatibility of graphene with inorganic components^18, 27–29^. On the other hand, graphene barely contribute to the capacity in operating potential windows of TiO_2_ (in the range of 1.0 between 3.0 V vs. Li^+^/Li). Therefore, it is highly desirable to develop other materials as supports for nanosized TiO_2_ deposition. Kim *et al*. reported that TiO_2_ nanoparticles were uniformly assembled onto high-conductivity indium tin oxide nanowire arrays, which exhibited a large capacity of more than 200 mA h g^−1^ at a 60 C rate^30^. Gu *et al*. reported that Ag nanowires/mesoporous TiO_2_ delivered a reversible capacity of ~160 mA h g^−1^ after 230 cycles at a current density of 1 C^31^.

In the present work, heterogeneous TiO_2_@Nb_2_O_5_ composites, in which TiO_2_ nanoparticles were evenly embedded on ultrathin Nb_2_O_5_ nanosheets, were successfully synthesized for the first time. Furthermore, this composite was used as an anode for LIBs and delivered high reversible capacities and superior rate capability.

## Results

**Figure 1:**
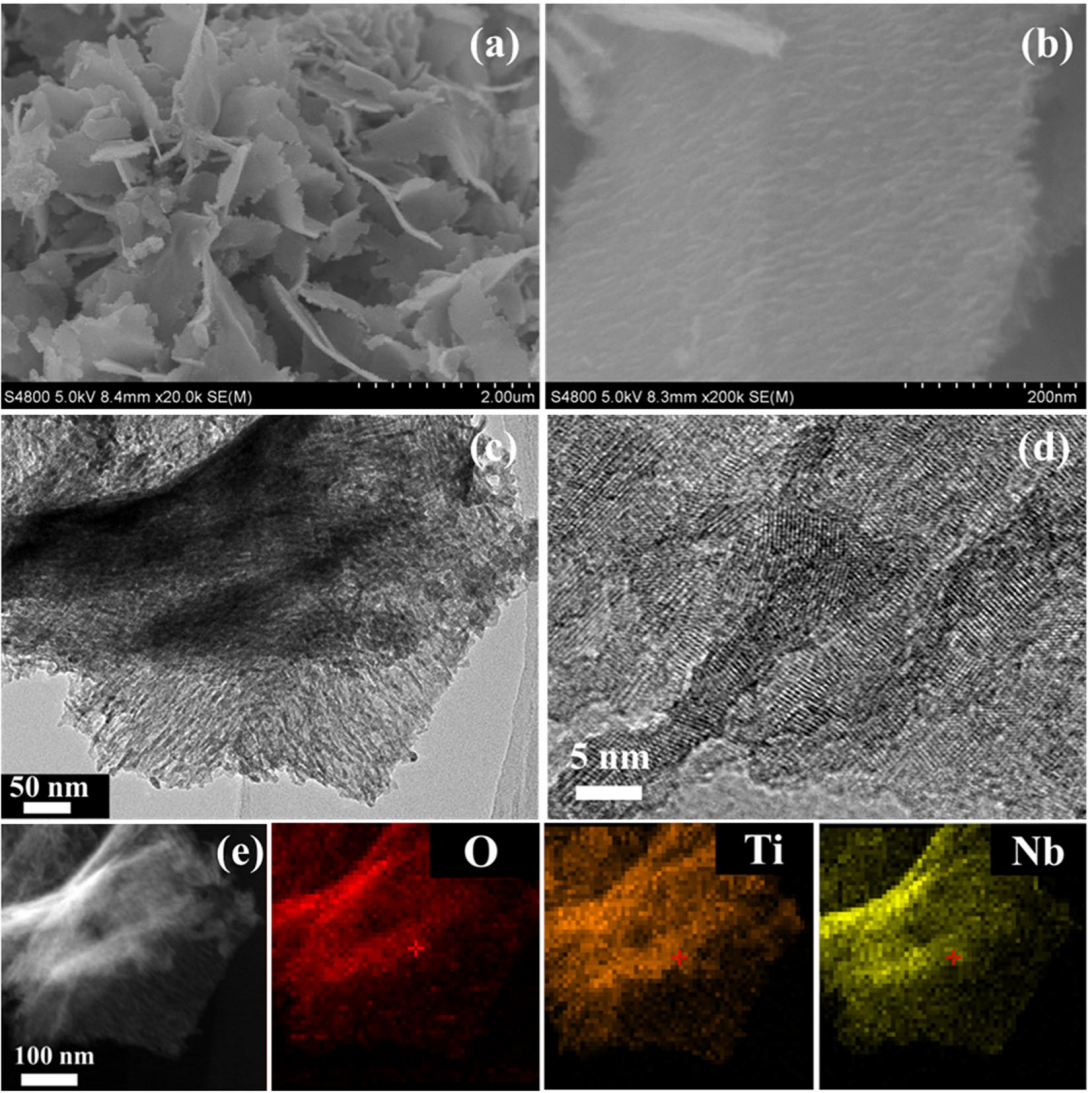
(**a**,**b**) SEM image, (**c**,**d**) TEM images and (**e**) STEM image and elemental mappings of TiO_2_@Nb_2_O_5_ composites.

The morphology and structure of TiO_2_@Nb_2_O_5_ composites are firstly characterized by SEM and TEM, respectively. A SEM image in Fig. 1a gives us a full view of the obtained TiO_2_@Nb_2_O_5_ composites, in which uniform sheet-like morphology with thin thickness can be observed. Unlike pure Nb_2_O_5_ nanosheets (Figure S1), the surface of TiO_2_@Nb_2_O_5_ composites appeared rough (Fig. 1b). It is noteworthy that the thickness of these nanosheets was larger than that of pure Nb_2_O_5_ nanosheets. The surface difference of TiO_2_@Nb_2_O_5_ composites and Nb_2_O_5_ nanosheets indicates that TiO_2_ nanoparticles were embedded on Nb_2_O_5_ nanosheets. Apart from the uniform TiO_2_@Nb_2_O_5_ composites, single TiO_2_ nanoparticles are not observed. TEM images in Fig. 1c shows that the whole surface of Nb_2_O_5_ nanosheets was covered with TiO_2_ nanoparticles, and further confirms that TiO_2_@Nb_2_O_5_ heterogeneous structure can be formed. In a HRTEM image of TiO_2_@Nb_2_O_5_ composites in Fig. 1d, it can be found that the size of TiO_2_ nanoparticles was about 5 nm, which is smaller than that of pure TiO_2_ nanoparticles (Figure S1f). To investigate the chemical composition of the TiO_2_@Nb_2_O_5_ composites, EDX analysis was carried out and the result is depicted in Figure S3. Strong O, Ti and Nb signals can be observed and ICP-OES results (Table S1) showed that the atomic ratio of Nb to Ti was 0.44. At the same time, STEM image and the corresponding elemental mappings O, Ti and Nb were performed to investigate the distribution of TiO_2_ nanoparticles in the Nb_2_O_5_ nanosheets. As shown in Fig. 1e, TiO_2_ nanoparticles were evenly embedded on the surface of Nb_2_O_5_ nanosheets.

**Figure 2:**
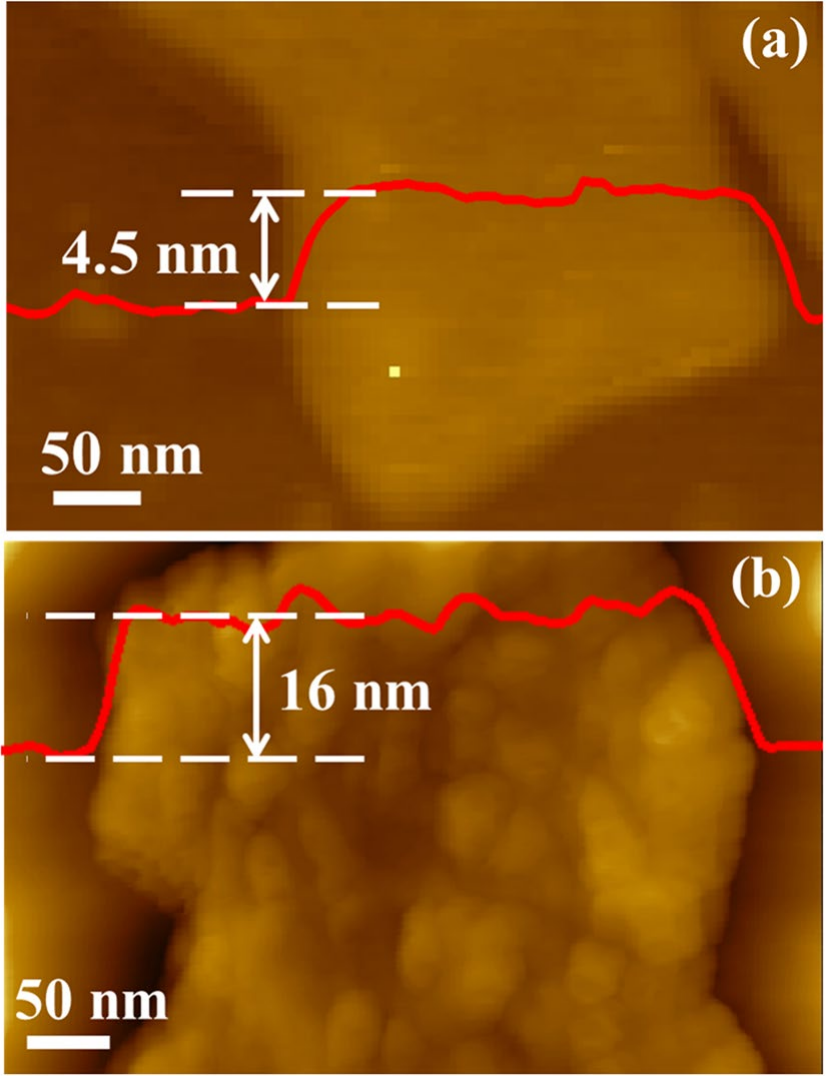
AFM images of (**a**) Nb_2_O_5_ nanosheets and (**b**) TiO_2_@Nb_2_O_5_ composites.

AFM measurement was performanced to determine the thicknesses of Nb_2_O_5_ nanosheets and TiO_2_@Nb_2_O_5_ composites. As shown in Fig. 2, the thicknesses of Nb_2_O_5_ nanosheets and TiO_2_@Nb_2_O_5_ composites were about 4.5 and 16 nm, respectively, further indicating that the size of TiO_2_ nanoparticles was about 6 nm. On the other hand, it can be clearly found that the surface of Nb_2_O_5_ nanosheets appeared smooth, while the surface of TiO_2_@Nb_2_O_5_ composites was rough. The AFM results were in good agreement with SEM and TEM results.

**Figure 3:**
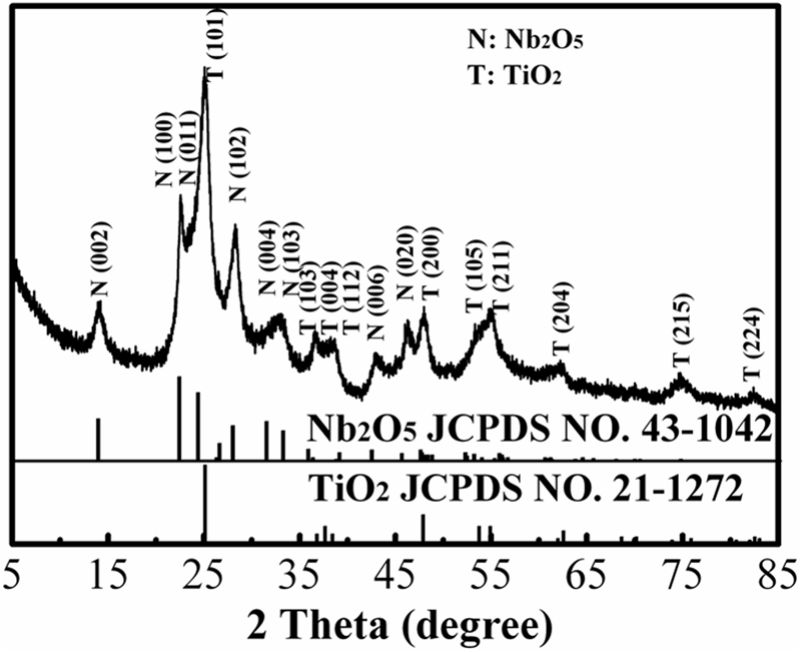
XRD patterns of TiO_2_@Nb_2_O_5_ composites.

Figure 3 shows the XRD patterns of TiO_2_@Nb_2_O_5_ composites. The diffraction peaks can be indexed to the mixed of monoclinic Nb_2_O_5_ (JCPDS 43-1042) and anatase TiO_2_ (JCPDS 21-1272). Furthermore, Raman spectra of TiO_2_ nanoparticles, Nb_2_O_5_ nanosheets and TiO_2_@Nb_2_O_5_ composites were also recorded and the results are presented in Figure S2. Raman spectrum of TiO_2_@Nb_2_O_5_ composites exhibited the characteristic peaks of anatase TiO_2_ and weak peaks of Nb_2_O_5_, which is consistent with the XRD result.

**Figure 4:**
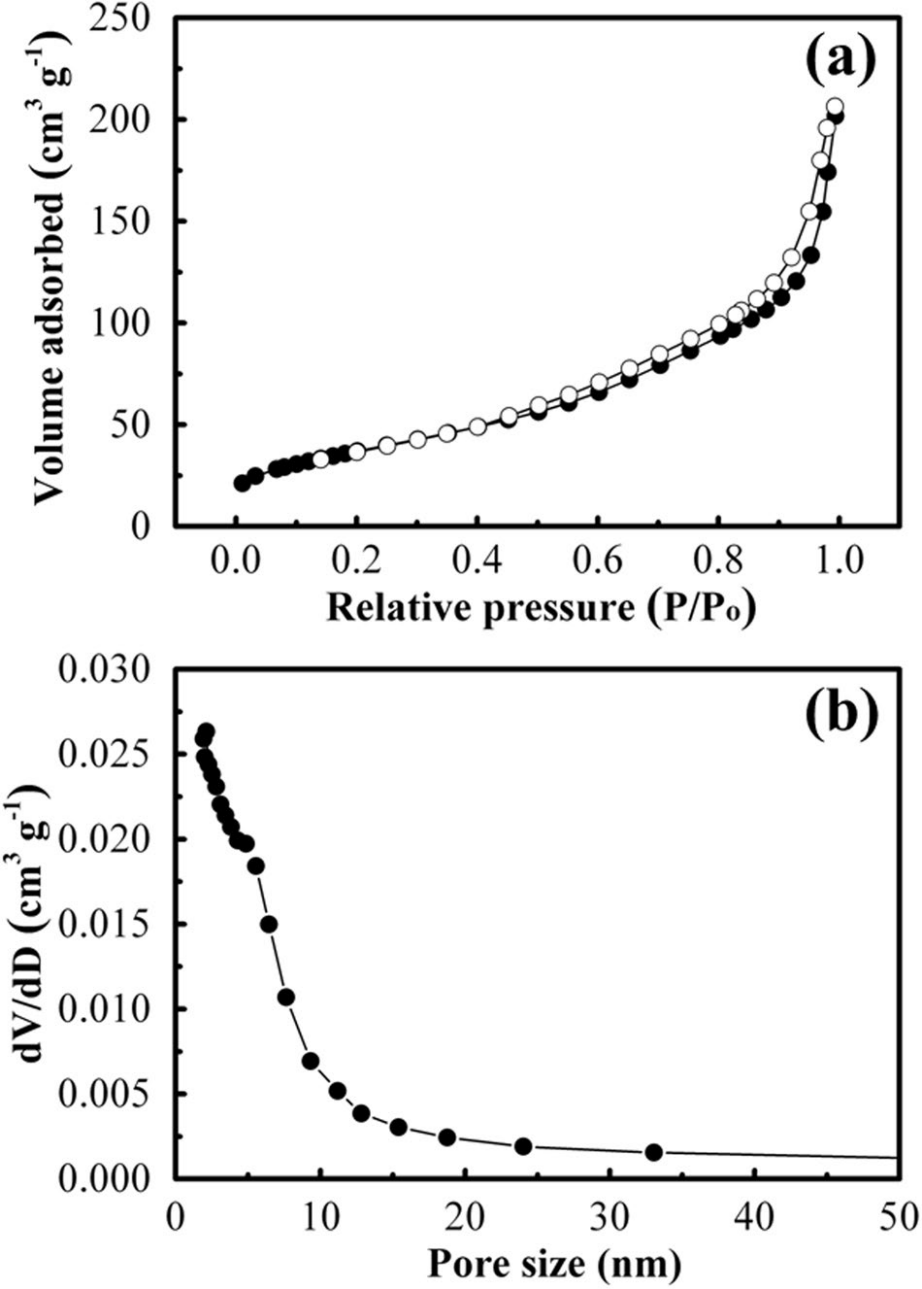
(**a**) N_2_ adsorption-desorption isotherms of TiO_2_@Nb_2_O_5_ composites and (**b**) the corresponding pore size distributions calculated using the BJH method.

To investigate the Brunauer-Emmett-Teller (BET) surface area and porous structure of TiO_2_@Nb_2_O_5_ composites, N_2_ adsorption-desorption isotherms of this material was measured and the results are depicted in Fig. 4. Similar to Nb_2_O_5_ nanosheets (Figure S5b), TiO_2_@Nb_2_O_5_ composites exhibited a type-IV isotherm with a type-H3 hysteresis loop, indicating that TiO_2_@Nb_2_O_5_ composites maintain sheet-like morphology. However, it is noteworthy that the range of hysteresis loop for TiO_2_@Nb_2_O_5_ composites is larger than that of Nb_2_O_5_ nanosheets (Figure S5b), which can be ascribed to the slight aggregation of TiO_2_ nanoparticles on the surface of Nb_2_O_5_ nanosheets. The BET surface area of TiO_2_ nanoparticles, Nb_2_O_5_ nanosheets and TiO_2_@Nb_2_O_5_ composites are 85.6, 99.8 and 134.6 m^2^ g^−1^, respectively. Homogeneous dispersion of TiO_2_ nanoparticles onto Nb_2_O_5_ nanosheets increased the thickness of these composites and inhibited aggregation of nanosheets. On the other hand, the structure of TiO_2_ nanoparticles embedded on nanosheets prevents the undesirable aggregation. Therefore, TiO_2_@Nb_2_O_5_ composites show an inconspicuous BJH pore size (Fig. 4b), which is different to that of Nb_2_O_5_ nanosheets (inset in Figure S5b) or TiO_2_ nanoparticles (inset in Figure S5a).

**Figure 5:**
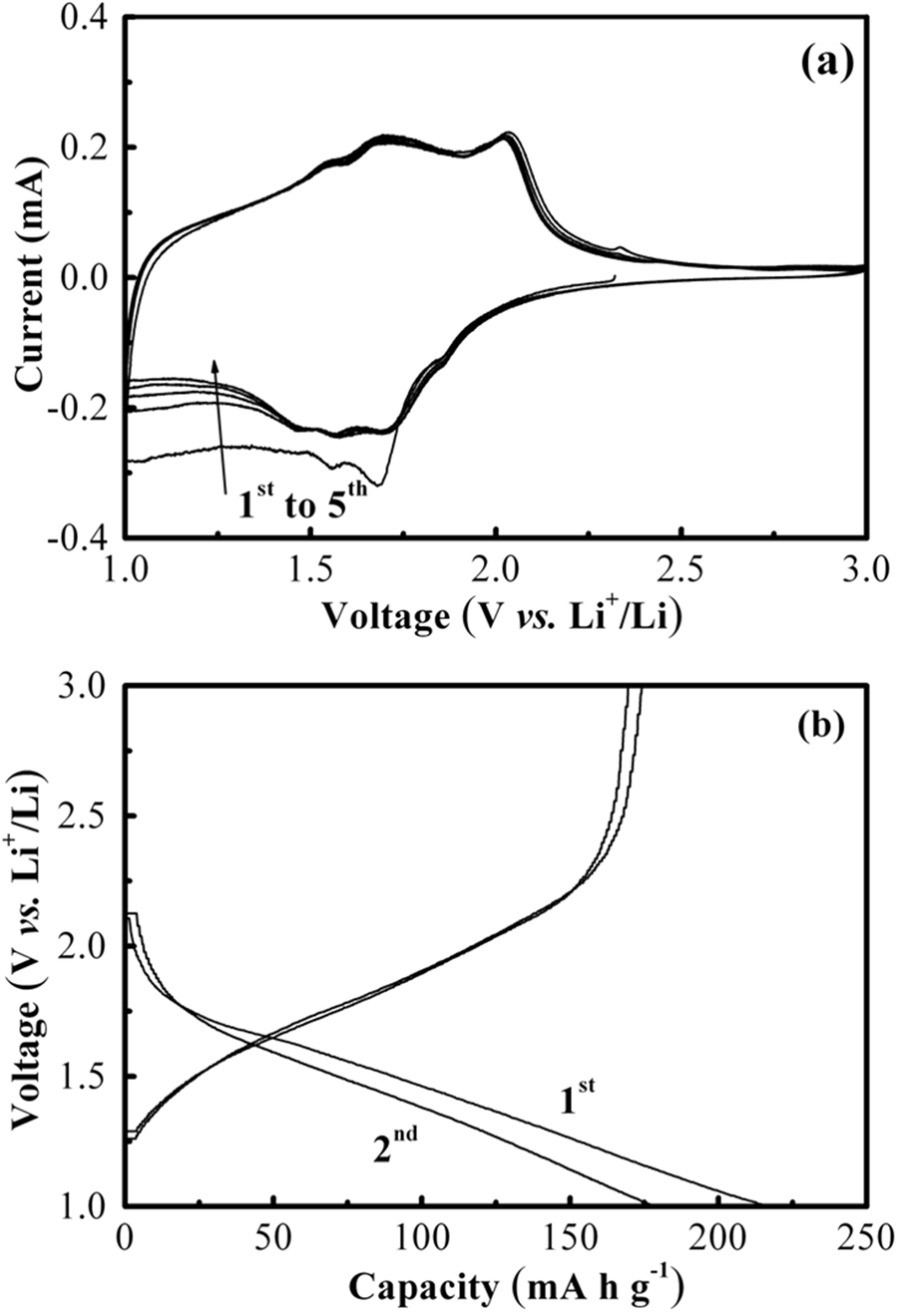
The electrochemical properties of TiO_2_@Nb_2_O_5_ composites: (**a**) CV curves with a scan rate of 0.5 mV s^−1^ and (**b**) charge-discharge profiles at a current density of 1 A g^−1^.

Figure 5a shows CV curves of TiO_2_@Nb_2_O_5_ composites at a scan rate of 0.5 mV s^−1^ in range of 1.0–3.0 V. All cathodic and anodic peaks can be ascribed to the mixed of monoclinic Nb_2_O_5_ and anatase TiO_2_. Several broad cathodic and anodic peaks in the potential range of 1.1–2.2 V can be observed, which are similar to those of other Nb_2_O_5_^32–36^. In addition, TiO_2_@Nb_2_O_5_ composites also shows representative CV curves of anatase TiO_2_, in which two well-defined cathodic and anodic peaks at ~1.7 and 2.0 V can be clearly observed, respectively. The electrochemical performance of TiO_2_@Nb_2_O_5_ composites was evaluated by galvanostatic charge-discharge cycling at different current densities. Fig. 5b shows the charge-discharge voltage profiles of TiO_2_@Nb_2_O_5_ composites electrode in the 1st and 2nd cycle at a current density of 1 A g^−1^. Interestingly, TiO_2_@Nb_2_O_5_ composites also displayed sloping charge-discharge profiles, which was very similar to that of Nb_2_O_5_ nanosheets (Figure S7a). Namely, the two typical voltage plateaus of TiO_2_ cannot be observed in charge-discharge voltage profiles of TiO_2_@Nb_2_O_5_ composites. This might be ascribed to the effect of particle size of TiO_2_ on the galvanostatic charge-discharge process^13^. Comparison with Nb_2_O_5_ nanosheets (Figure S7a) or TiO_2_ nanoparticles (Figure S7b), TiO_2_@Nb_2_O_5_ composites delivered a superior initial discharge and charge capacities of 216.8 and 174.3 mA h g^−1^ with a high Coulombic efficiency of 80.4%. The first irreversible capacity loss can be attributed to some irreversible side reactions inside the electrode materials^37^.

**Figure 6:**
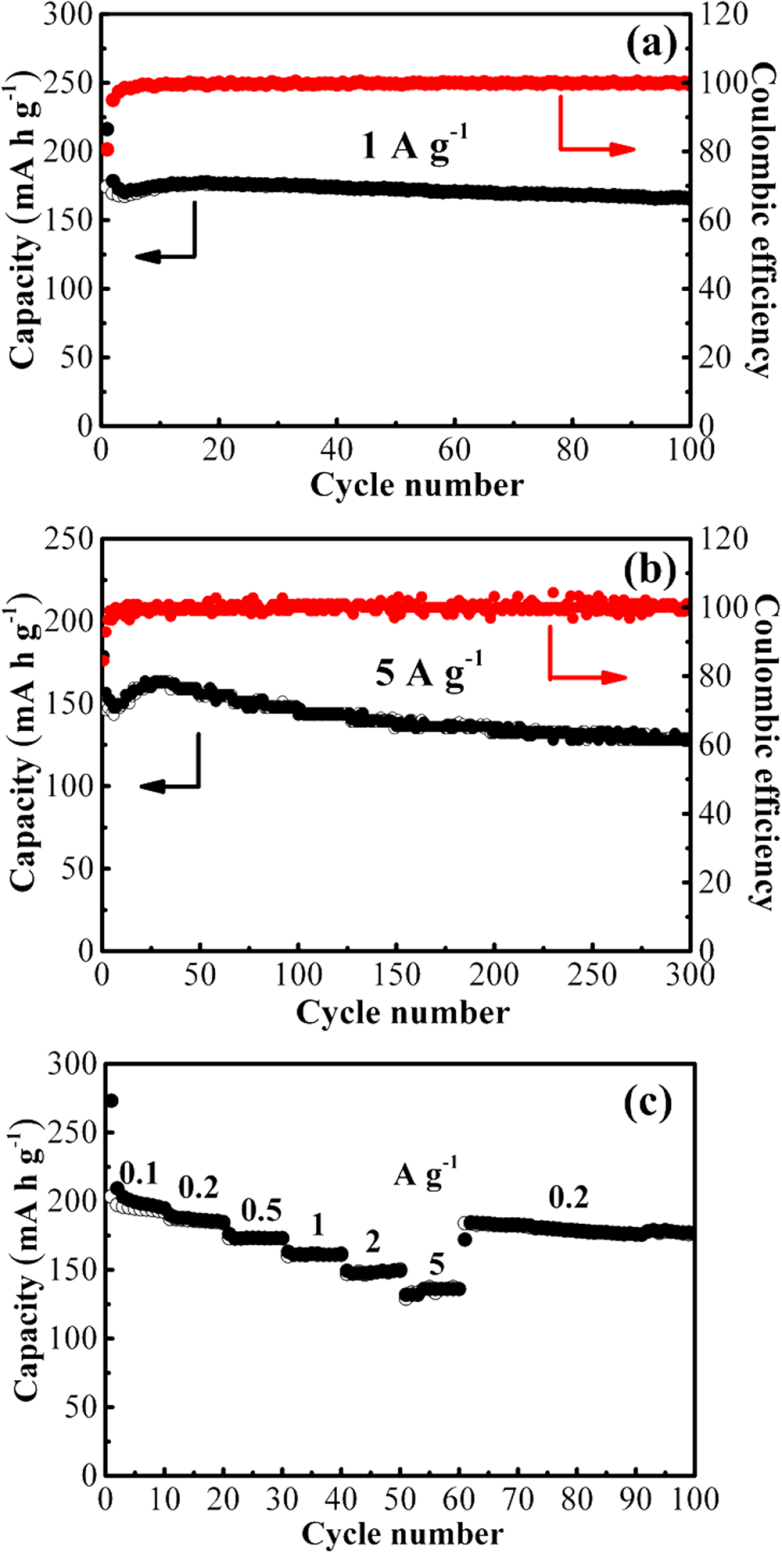
The electrochemical properties of TiO_2_@Nb_2_O_5_ composites: cycling performances at different current densities of (**a**) 1 and (**b**) 5 A g^−1^ and (**c**) rate capability from 0.1 to 5 A g^−1^.

As shown in Fig. 6, TiO_2_@Nb_2_O_5_ composites exhibited high reversible capacities of 166.3 mA h g^−1^ after 100 cycles at 1 A g^−1^ and 129.1 mA h g^−1^ after 300 cycles at 5 A g^−1^, respectively. In addition, the Coulombic efficiency rapidly increased to nearly 100% after first several cycles. On the other hand, the rate capability of TiO_2_ nanoparticles, Nb_2_O_5_ nanosheets and TiO_2_@Nb_2_O_5_ composites were evaluated and the results were shown in Fig. 6c and Figure S9. It is clear that TiO_2_@Nb_2_O_5_ composites exhibited a significantly improved rate capability over TiO_2_ nanoparticles or Nb_2_O_5_ nanosheets. The capacities of 194.9, 184.8, 173.2, 161.8, 149.3 and 136.0 mA h g^−1^ were achieved for TiO_2_@Nb_2_O_5_ composites at 0.1, 0.2, 0.5, 1, 2 and 5 A g^−1^, respectively. When the current density was back to 0.2 A g^−1^, the capacity of 177.1 mA h g^−1^ was obtained after 50 cycles.

**Figure 7:**
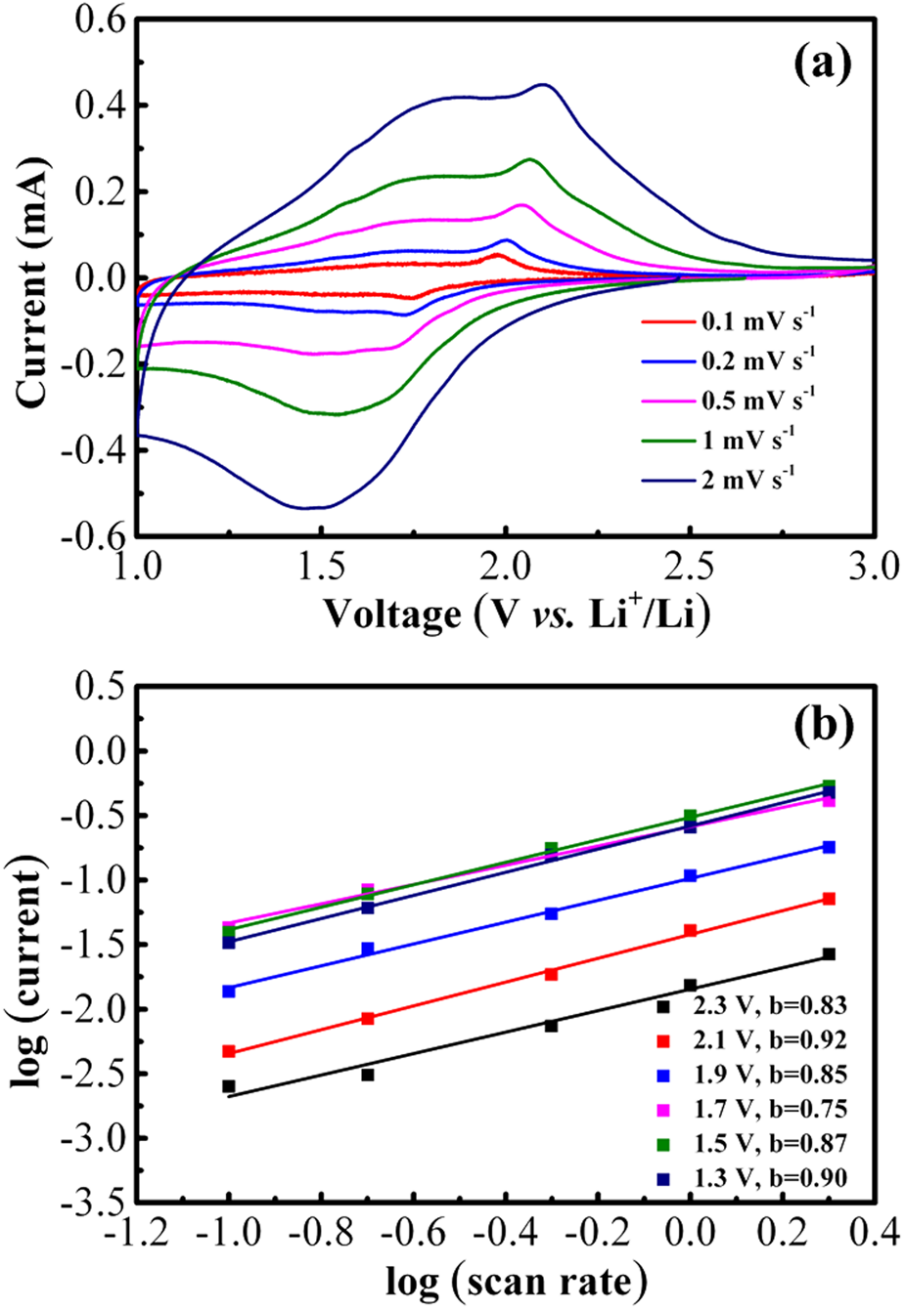
(**a**) CV curves of TiO_2_@Nb_2_O_5_ composites with different scan rates and (**b**) their corresponding log (current) *vs* log (scan rate) fitting lines.

It is well known that Nb_2_O_5_ is a kind of pseudocapacitative materials^32–34^. At the same time, TiO_2_ with small size also shows a pseudocapacitative behavior^12, 13^. To further authenticate the pseudocapacitive feature in TiO_2_@Nb_2_O_5_ composites, CV curves with different scan rates in cathodic process were presented in Fig. 7a. The total stored charge can be separated into three componets: the Faradaic contribution from the Li^+^ ion intercalation process, pseudocapacitance and nonFaradaic contribution from the double layer effect. The differnet storage mechanisms can be determined by investigating the change of the peak current (i) with the scan rate (ν) according to the following equation 1 ^13^:

1 $${\rm{i}}={{\rm{a}}{\rm{\nu }}}^{{\rm{b}}}$$

where both a and b are adjustable parameters. When the b value is about 0.5, it implies that Li^+^ ion intercalation process is a dominant process; while the b value is close to 1, it indicates that stored charge mostly come from the surface capacitive effect. As shown in Fig. 7b, the fitting b values at the voltages of 1.3, 1.5, 1.9, 2.1 and 2.3 V were 0.90, 0.87, 0.85, 0.92 and 0.83, respectively, which are very close to 1 and suggest that the lithium storage process is mostly dominated by the pseudocapacitative contributions. It’s worth noting that the fitting b value of 0.75 at the voltage of 1.7 V was relatively low, which implied the current comes primarily from Li^+^ ion intercalation and pseudocapacitative process. The high performance of TiO_2_@Nb_2_O_5_ composites would be mainly related to this pseudocapacitive storage process.

## Discussion

In the present work, heterogeneous TiO_2_@Nb_2_O_5_ composites show high capacities at high-rate over TiO_2_@carbonaceous materials, as listed in Table 1. The excellent performance of TiO_2_@Nb_2_O_5_ composites can be attributed to the synergistic effects of nanostructured TiO_2_ and Nb_2_O_5_ as following: (i) TiO_2_@Nb_2_O_5_ composites with a surface area as large as 134.6 m^2^ g^−1^ can enhance the contact between the electrolyte and the electrode; (ii) nanosheet-like Nb_2_O_5_ as a support can buffer aggregation of TiO_2_ nanoparticles, and TiO_2_ nanoparticles embedded on Nb_2_O_5_ nanosheets offer a short diffusion distance and more surface storage sites for Li^+^ ions, thus promoting fast and reversible lithium intercalation/deintercalation into/from electrode and enhanced capacity; (iii) pseudocapacitive behavior of Nb_2_O_5_ nanosheets can deliver a high reversible capacity at high current densities^32, 33, 38^.

**Table 1:** Comparison of the cycling performance of TiO_2_@Nb_2_O_5_ with some representative TiO_2_@carbonaceous materials as an anode for LIBs.

Composite TiO_2_	Discharge capacity (mA h g^−1^)	Current density (mA g^−1^)	References
TiO_2_@Nb_2_O_5_	166.3 (after 100 cycles)	1000	present work
	129.1 (after 300 cycles)	5000	
TiO_2_@carbon nanotube	203 (after 100 cycles)	100	16
	101 (after 100 cycles)	4000	
TiO_2_ nanosheets@graphene	161 (after 120 cycles)	170	19
	119 (after 120 cycles)	1700	
TiO_2_ nanoparticles@graphene	175 (after 100 cycles)	100	20
	125 (after 100 cycles)	2000	
TiO_2_ quantum-dot@graphene	190 (after 100 cycles)	168	24
	145 (after 100 cycles)	1680	
TiO_2_@mesoporous carbon	133.9 (after 100 cycles)	500	39
	81.2 (after 100 cycles)	3000	

In summary, a two-step synthetic route was firstly developed for synthesizing heterogeneous TiO_2_@Nb_2_O_5_ composites, in which TiO_2_ nanoparticles were evenly embedded on the surface of ultrathin Nb_2_O_5_ nanosheets. When used as anode material for lithium-ion batteries, it showed high capacities and superior rate capability in comparison to pure TiO_2_ nanoparticles or Nb_2_O_5_ nanosheets due to their synergistic effects as following: The composite with a large surface area can enhance the contact between the active material and the electrolyte; the aggregation of TiO_2_ nanoparticles can be buffered on the surface of Nb_2_O_5_ nanosheets; TiO_2_ nanoparticles embedded on Nb_2_O_5_ nanosheets offer a short diffusion distance and more surface storage sites for Li^+^ ions; pseudocapacitive behavior of Nb_2_O_5_ can deliver a high reversible capacity at high current densities. Therefore, such heterogeneous nanostructure has a great potential application in field of photocatalysis, Li/Na-ion batteries and supercapacitors.

## Methods

### Synthesis of Nb_2_O_5_ nanosheets, TiO_2_ nanoparticles and TiO_2_@Nb_2_O_5_ composites

6 g of urea was dissolved in 30 mL of ethylene glycol (EG). After stirring for 10 min, 0.25 g of niobium (V) oxalate hydrate was added into above solution under stirring, and then the resulting solution was transferred into Teflon coated stainless steel with a capacity of 50 mL. The autoclave was kept at 200 °C for 2 days and then naturally cooled to room temperature. The white product was harvested via centrifugation, washed thoroughly with ethanol for several times and dried in an oven at 70 °C overnight. To obtain Nb_2_O_5_ nanosheets, the above white product was annealed at 500 °C in air for 2 h with a heating rate of 2 °C min^−1^.

For the synthesis of TiO_2_@Nb_2_O_5_ composites, the 50 mg of as-prepared Nb_2_O_5_ nanosheets were added into a 50 mL Teflon container with pre-filled with 30 mL ethylene glycol (EG) under stirring. After stirring for 3 h, 0.2 mL of titanium isopropoxide (TTIP) was dropwise added into the above suspension. After stirring for another 3 h, 4.0 g of urea was dissolved in above solution under stirring to form a white solution which was transferred into Teflon coated stainless steel. The autoclave was kept at 180 °C for 24 h and naturally cooled to room temperature. The white product was separated by centrifugation, washed with ethanol for several times and dried in an oven at 70 °C overnight. The resulting product was annealed at 400 °C in air for 2 h with a heating rate of 2 °C min^−1^ and TiO_2_@Nb_2_O_5_ composites were obtained.

TiO_2_ nanoparticles were synthesized following the same procedure of TiO_2_@Nb_2_O_5_ composites except for the addition of Nb_2_O_5_ nanosheets.

### Characterizations

X-ray diffraction (XRD) patterns of products were recorded on a Rigaku Ultima IV X-ray diffractomator by using CuKα radiation. Scanning electron microscopy (SEM, Hitachi S4800 instrument) and transmission electron microscopy (TEM, FEI F20 S-TWIN instrument) were applied for the determination of products morphology and composition. The STEM mapping is TEM-based STEM and a voltage of 200 KV was used for the mapping. Atomic Force Microscope (AFM, Agilent Technologies) was used to determine the thickness and morphology of products. The Raman spectra were recorded in a Renishaw inVai Raman microscope with a 532 nm laser. N_2_ adsorption-desorption was performed on a Micromeritics ASAP 2020 instrument (Micromeritics, Norcross, GA, USA). BET surface area of the obtained samples were measure by nitrogen adsorption and desorption isotherms at 77 K after the samples were degassed under vacuum at 220 °C for 6 h. The pore size distributions of the samples were analyzed by using the BJH methods. The contents of Nb_2_O_5_ and TiO_2_ in the prepared composites were determined by PerkinElmer Optima 8000 inductively coupled plasma optical emission spectrometry (ICP-OES).

### Electrochemical measurements

The electrochemical performance of all products was performed using 2025-type coin cells with two-electrodes. First, the resulting products were admixed with polyvinylidene fluoride (PVDF) binder and acetylene black carbon in a weight ratio of 7:2:1 to form a slurry which was coated on copper foil circular flakes and dried at 110 °C in a vacuum overnight. Copper foils coated active materials were used as working electrodes and Lithium foils were used as the counter electrodes. The electrolyte was 1 M LiPF_6_ in a 1:1:1 (volume ratio) mixture of ethylene carbonate (EC), ethylene methyl carbonate (EMC) and dimethyl carbonate (DMC). Celgard2400 (America) microporous polypropylene membrane was used as the separator. Cell assembly was carried out in a glove box filled with highly pure argon gas (O_2_ and H_2_O levels <1 ppm). Cyclic voltammetry (CV) and charge-discharge tests of all electrodes were performed using an electrochemical workstation (CHI 600 C) and Land automatic batteries tester (Land, CT 2001A, Wuhan, China), respectively. The specific capacities of TiO_2_@Nb_2_O_5_ were calculated based on the weight of the composites.

## Electronic supplementary material

Supplementary Information
