# Supplementary material for: Heterogeneous TiO2@Nb2O5 composite as a high-performance anode for lithium-ion batteries
Source: Sci Rep. 2017 Aug 3;7:7204. doi: 10.1038/s41598-017-07562-5 (PMC5543047; doi:10.1038/s41598-017-07562-5)
Supplement: Supplementary file 1 — Supplementary Information [file 41598_2017_7562_MOESM1_ESM.doc]

**Supplementary Information**

**Heterogeneous TiO_2_@Nb_2_O_5_ composite as a high-performance anode for lithium-ion batteries**

Yubin Liu^1,2^, Liwei Lin^2^, Weifeng Zhang^1,2^ and Mingdeng Wei^1,2^,*

1 State Key Laboratory of Photocatalysis on Energy and Environment, Fuzhou University, Fuzhou, Fujian 350002, China;

2 Institute of Advanced Energy Materials, Fuzhou University, Fuzhou, Fujian 350002, China;

*E-mail: [wei-mingdeng@fzu.edu.cn](mailto:wei-mingdeng@fzu.edu.cn); Tel/Fax: 86 591 83753180.


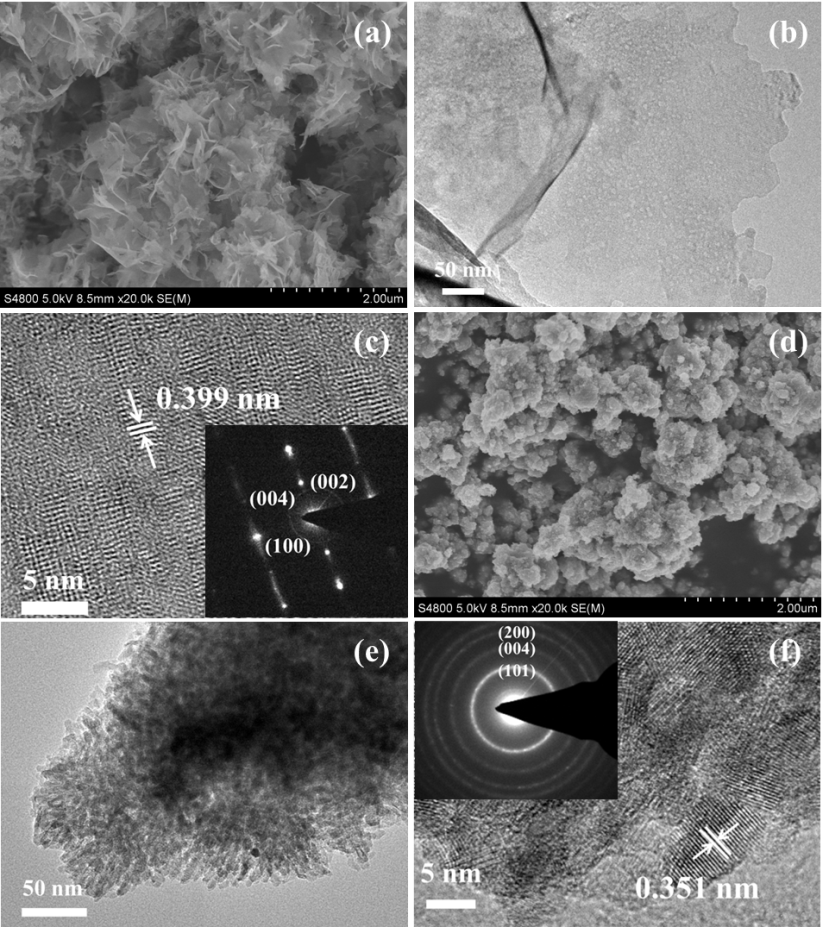


**Figure S1.** (a) SEM image and (b-c) TEM images of Nb_2_O_5_ nanosheets; (d) SEM image and (e-f) TEM images of TiO_2_ nanoparticles. The insets in (c and f) are the corresponding SAED patterns.

Figure S1a shows SEM image of Nb_2_O_5_, in which uniform ultrathin Nb_2_O_5_ nanosheets with high elasticity can be observed. It is noteworthy that the surface of Nb_2_O_5_ nanosheets appears smooth. TEM image of Nb_2_O_5_ nanosheets presented in Figure S1b reveals that the thickness of Nb_2_O_5_ nanosheets was several nanometers. Such nanosheets with a high structural flexibility were generally considered as an excellent support for other electrode materials. From HRTEM image in Figure S1c, it can be found that the lattice fringe was approximately 0.399 nm, corresponding to the d_100_ spacing of monoclinic Nb_2_O_5_ (JCPDS 43-1042). A selected-area electron diffraction (SAED) pattern (inset in Figure 2c) exhibits sharp diffraction spots, indicating that Nb_2_O_5_ nanosheets presented a single crystalline characteristic. On the other hand, the morphology and structure of pure TiO_2_ were also defined. Figure S1d shows that a large number of TiO_2_ nanoparticles were aggregated severely. The porous structure can be clearly observed in TEM image in Figure S1e. A HRTEM image in Figure S1f shows that the size of TiO_2_ nanoparticles was approximately 9 nm with highly crystalline. The lattice fringe was about 0.351 nm, corresponding to the d_101_ spacing of anatase TiO_2_ (JCPDS 21-1272). At the same time, a corresponding SAED pattern (inset in Figure 1Sf) reveals a set diffraction rings, which can be indexed to the (101), (004) and (200) planes of anatase TiO_2_ (JCPDS 21-1272).


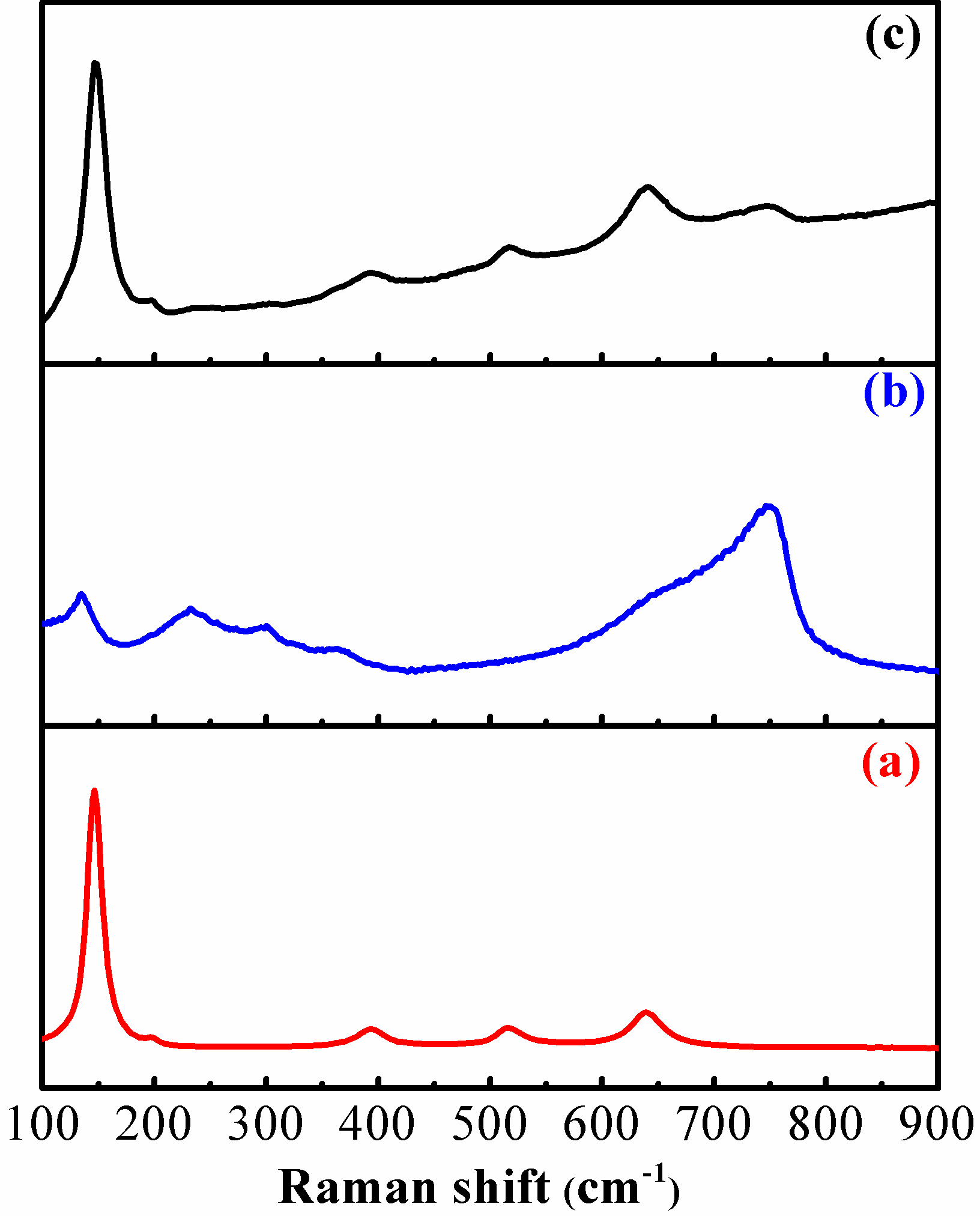


**Figure S2.** Raman spectra of (a) TiO_2_ nanoparticles, (b) Nb_2_O_5_ nanosheets and (c) TiO_2_@Nb_2_O_5_ composites.


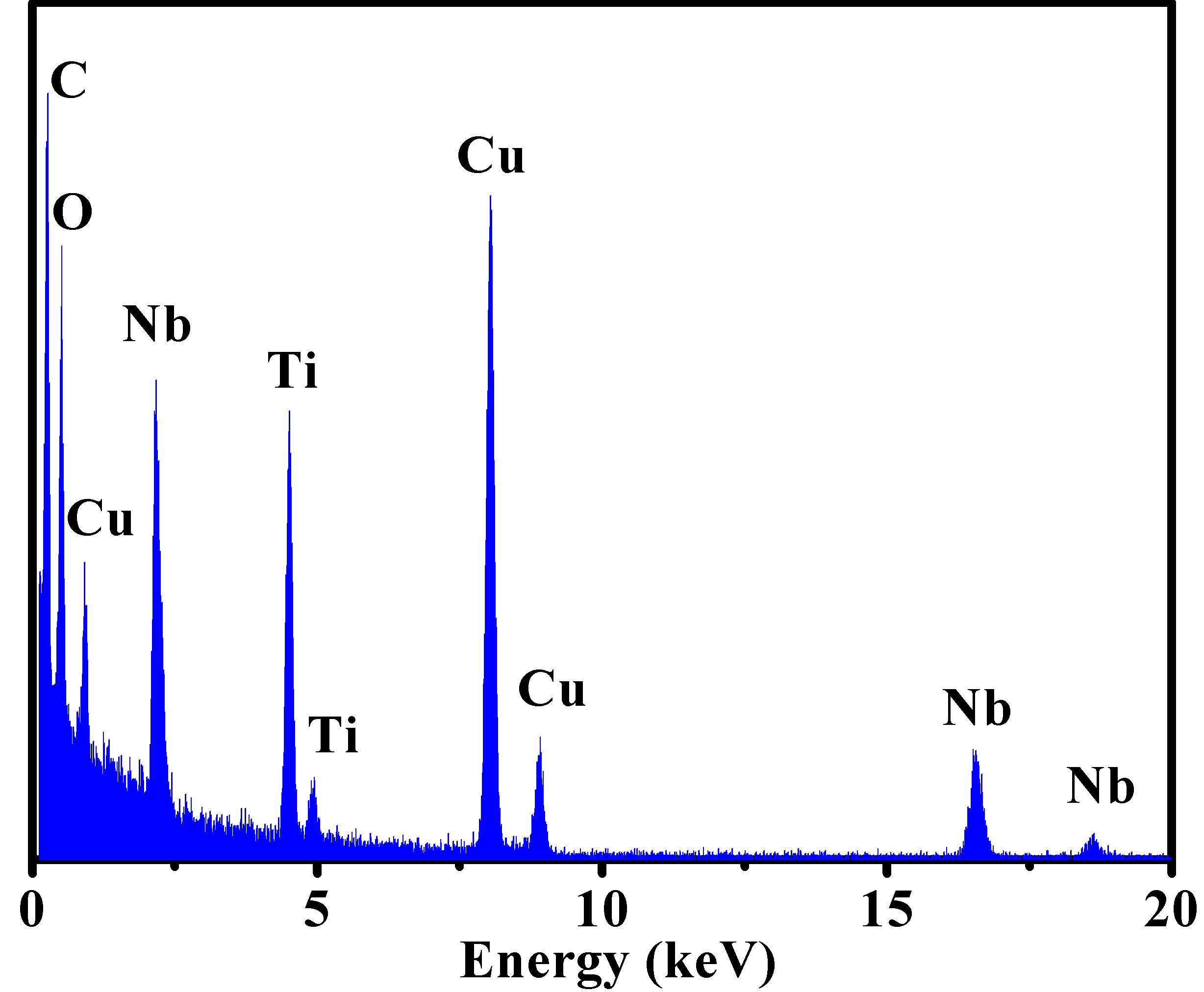


**Figure S3.** EDX spectra of TiO_2_@Nb_2_O_5_ composites.

**Table S1** Stoichiometry calculations of the chemical compositions of the prepared composites from ICP-OES analysis

| Samples | Atomic ratio of Nb to Ti | Weight ratio of Nb_2_O_5_ to TiO_2_ |
| --- | --- | --- |
| TiO_2_@Nb_2_O_5_-0.1 | 1.20 | 2.01 |
| TiO_2_@Nb_2_O_5_ | 0.44 | 0.73 |
| TiO_2_@Nb_2_O_5_-0.4 | 0.14 | 0.23 |


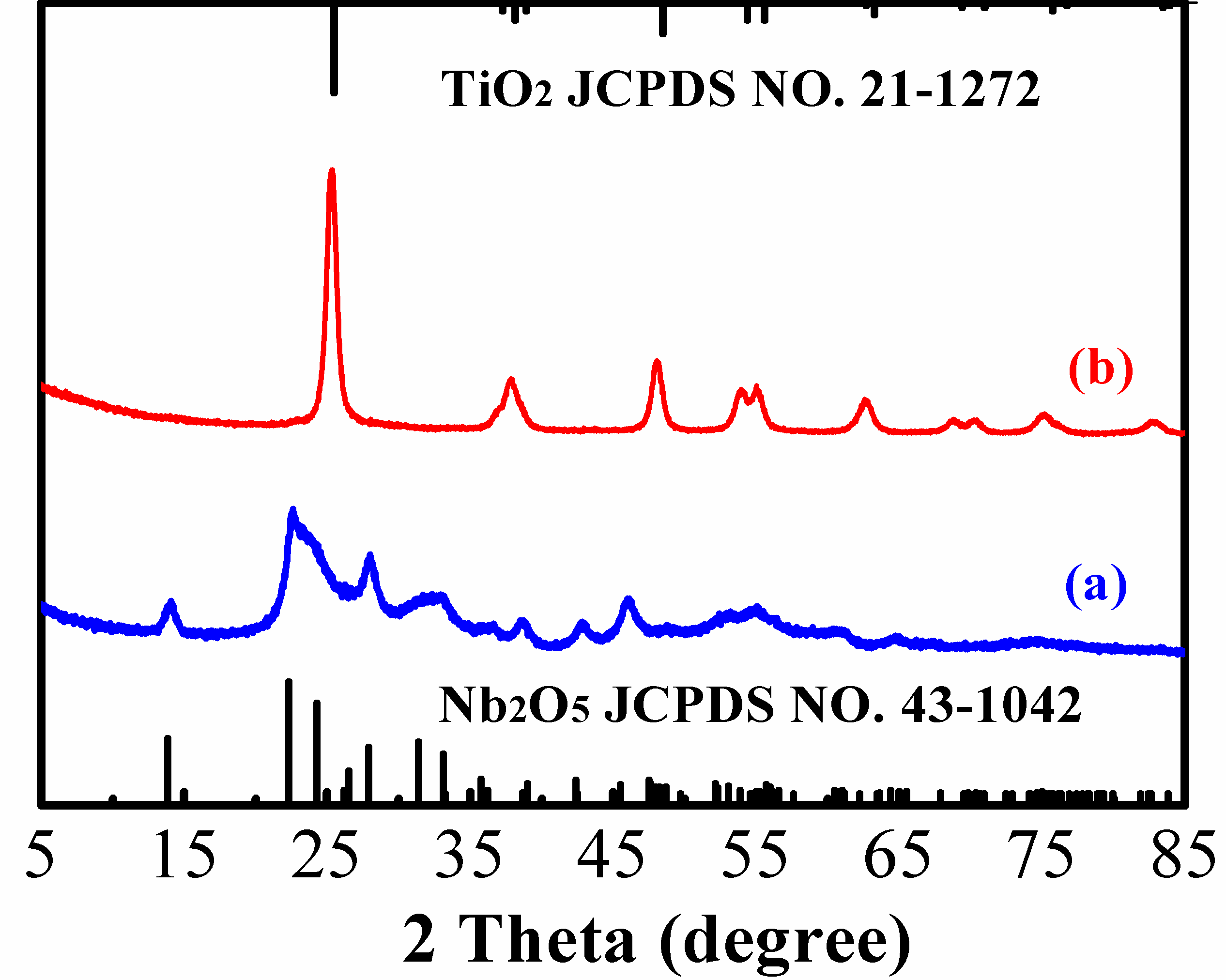


**Figure S4.** XRD patterns of (a) Nb_2_O_5_ nanosheets and (b) TiO_2_ nanoparticles.

Figure S4 shows the XRD patterns of Nb_2_O_5_ nanosheets and TiO_2_ nanoparticles. All diffraction peaks in TiO_2_ nanoparticles and Nb_2_O_5_ nanosheets are consistent with those of pure anatase TiO_2_ (JCPDS 21-1272) and pure monoclinic Nb_2_O_5_ (JCPDS 43-1042), respectively.


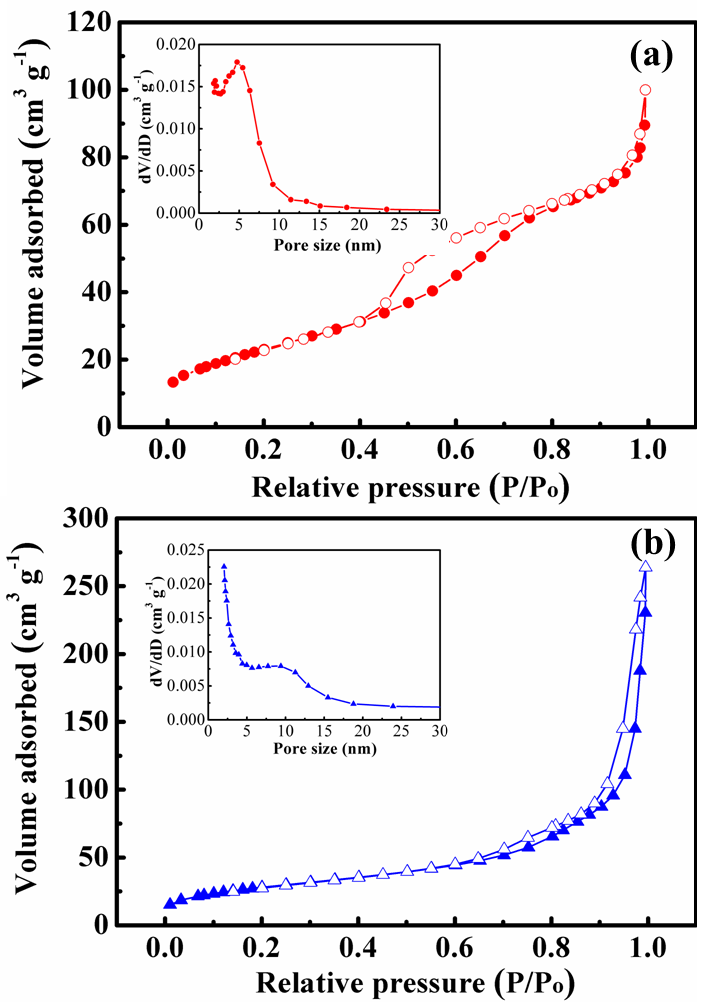


**Figure S5.** N_2_ adsorption-desorption isotherms of (a) TiO_2_ nanoparticles and (b) Nb_2_O_5_ nanosheets. The insets in (a and b) are the corresponding pore size distributions calculated using the BJH method.

As shown in Figure S5a, pure TiO_2_ nanoparticles exhibited a type-IV isotherm with a type-H2 hysteresis loop, which can be ascribed to aggregation of particles forming ink-bottle pores. Nb_2_O_5_ nanosheets exhibited a type-IV isotherm with a type-H3 hysteresis loop, which reveals that Nb_2_O_5_ nanosheets are composed of aggregation of sheet-like particles forming slit-like pores ^1^. A narrow Barretl-Joyner-Halenda (BJH) pore size of about 5 nm indicates that a uniform mesopore existed in TiO_2_ nanoparticles. Nb_2_O_5_ nanosheets show a broad BJH pore size of about 10 nm.


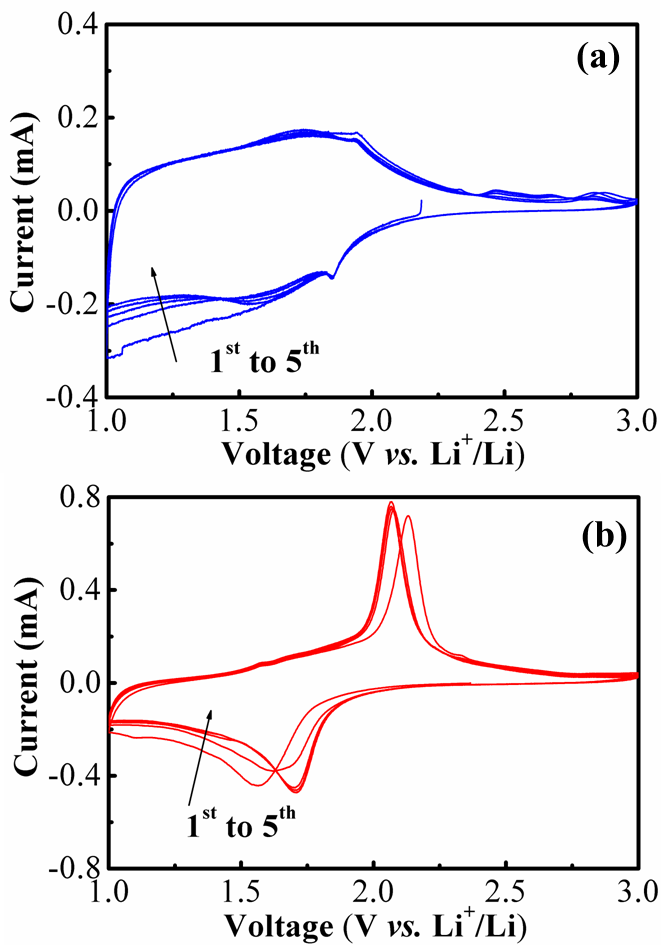


**Figure S6.** CV curves of (a) Nb_2_O_5_ nanosheets and (b) TiO_2_ nanoparticles with a scan rate of 0.5 mV s^-1^.

Figure S6 shows CV curves of Nb_2_O_5_ nanosheets and TiO_2_ nanoparticles at a scan rate of 0.5 mV s^-1^ in range of 1.0-3.0 V. The CV curves of Nb_2_O_5_ nanosheets in Figure S6a show several broad cathodic and anodic peaks in the potential range of 1.1-2.2 V. Figure S6b shows representative CV curves of anatase TiO_2_, in which two well-defined cathodic and anodic peaks at ~1.7 and 2.0 V can be clearly observed, respectively.


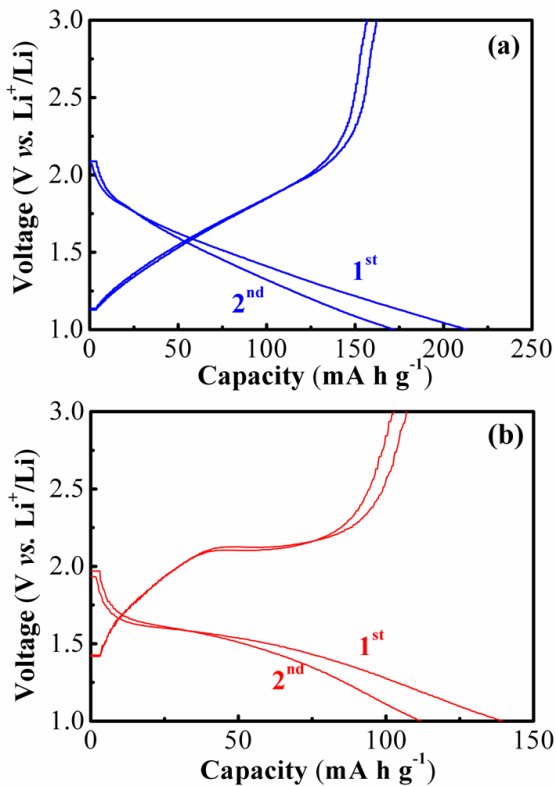


**Figure S7.** Charge-discharge profiles of (a) Nb_2_O_5_ nanosheets and (b) TiO_2_ nanoparticles at a current density of 1 A g^-1^.

Figure S7 shows the charge-discharge voltage profiles of TiO_2_ nanoparticles and Nb_2_O_5_ nanosheets electrodes in the 1st and 2nd cycle at a current density of 1 A g^-1^. It can be clearly seen that Nb_2_O_5_ nanosheets exhibited sloping charge-discharge profiles, indicating a single phase reaction ^2^. The initial discharge process displayed a high capacity of 213.3 mA h g^-1^ and a subsequent charge capacity of 162.0 mA h g^-1^. In Figure S7b, the pure TiO_2_ nanoparticles electrode showed a typical charge-discharge voltage profile. Two voltage plateaus at ~ 1.7 and 2.1 V can be observed during the discharging and charging processes.


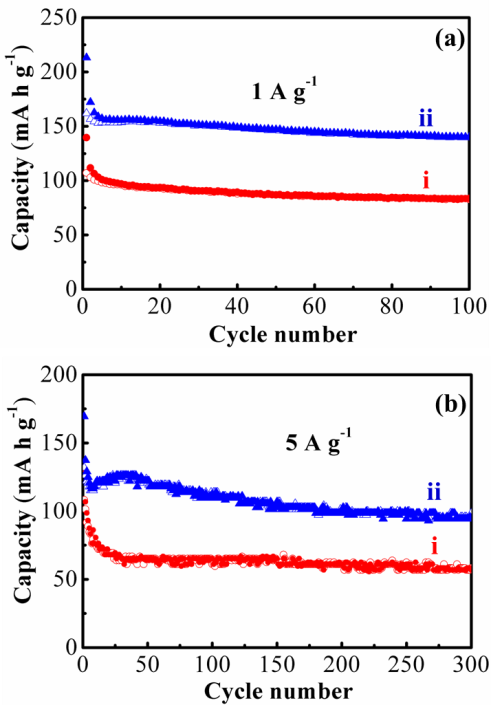


**Figure S8.** Cycling performances of TiO_2_ nanoparticles (i) and Nb_2_O_5_ nanosheets (ii) at different current densities of (a) 1 and (b) 5 A g^-1^.

As shown in Figure S8, TiO_2_ nanoparticles exhibited capacities of 83.6 mA h g^-1^ after 100 cycles at 1 A g^-1^ and 56.9 mA h g^-1^ after 300 cycles at 5 A g^-1^. Nb_2_O_5_ nanosheets displayed capacities of 139.8 mA h g^-1^ after 100 cycles at 1 A g^-1^ and 94.4 mA h g^-1^ after 300 cycles at 5 A g^-1^.


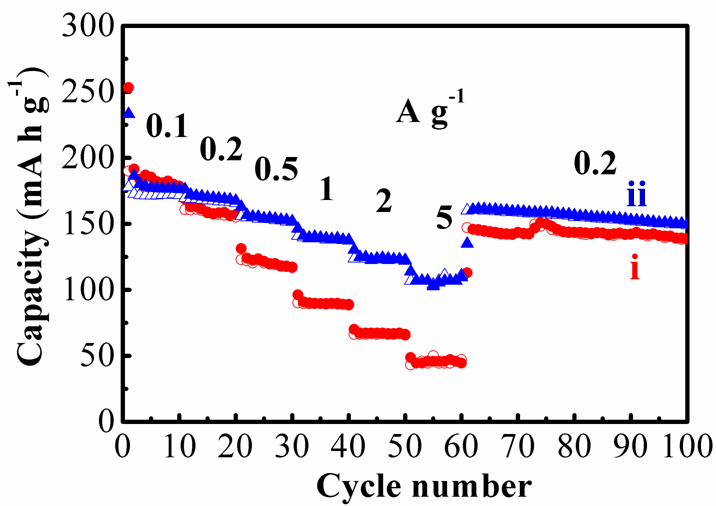


**Figure S9.** Rate capability of TiO_2_ nanoparticles (i) and Nb_2_O_5_ nanosheets (ii) from 1 to 5 A g^-1^.


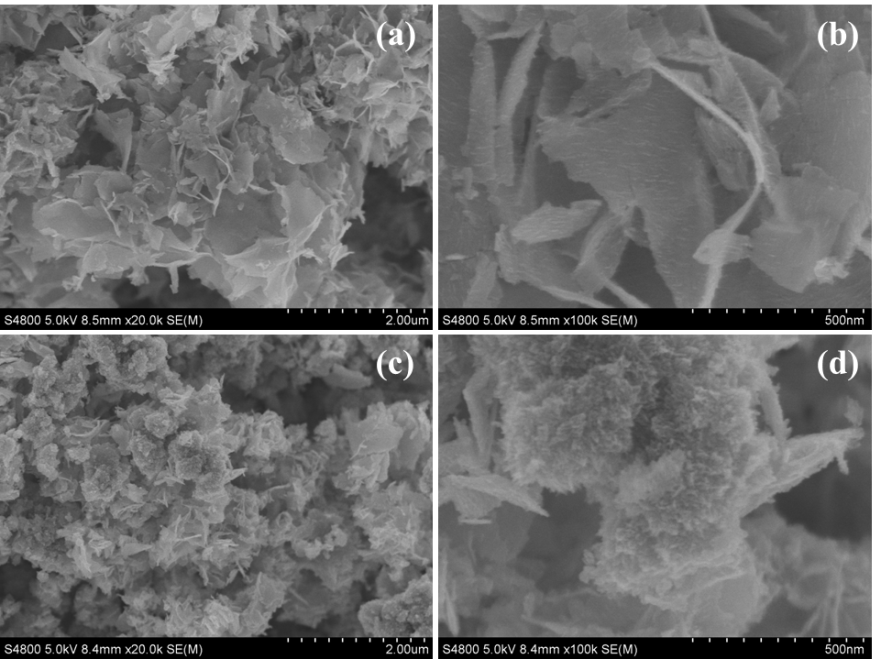


**Figure S10.** SEM images of (a-b) TiO_2_@Nb_2_O_5_-0.1 composites and (c-d) TiO_2_@Nb_2_O_5_-0.4 composites.

To investigate the optimized ratio of TiO_2_ nanoparticles and Nb_2_O_5_ nanosheets, the morphology of TiO_2_@Nb_2_O_5_-0.1 and TiO_2_@Nb_2_O_5_-0.4 composites were characterized by SEM and shown in Figure S10. Unlike rough TiO_2_@Nb_2_O_5_ composites, the surface of TiO_2_@Nb_2_O_5_-0.1 composites obtained with 0.1 mL TTIP appears smoother. When the volume of TTIP was increased to 0.4 mL, Nb_2_O_5_ nanosheets cannot offer more sites for TiO_2_ nanoparticles to embed. Therefore, redundant TiO_2_ nanoparticles were aggregated, which can be clearly observed in Figure S10c-d.


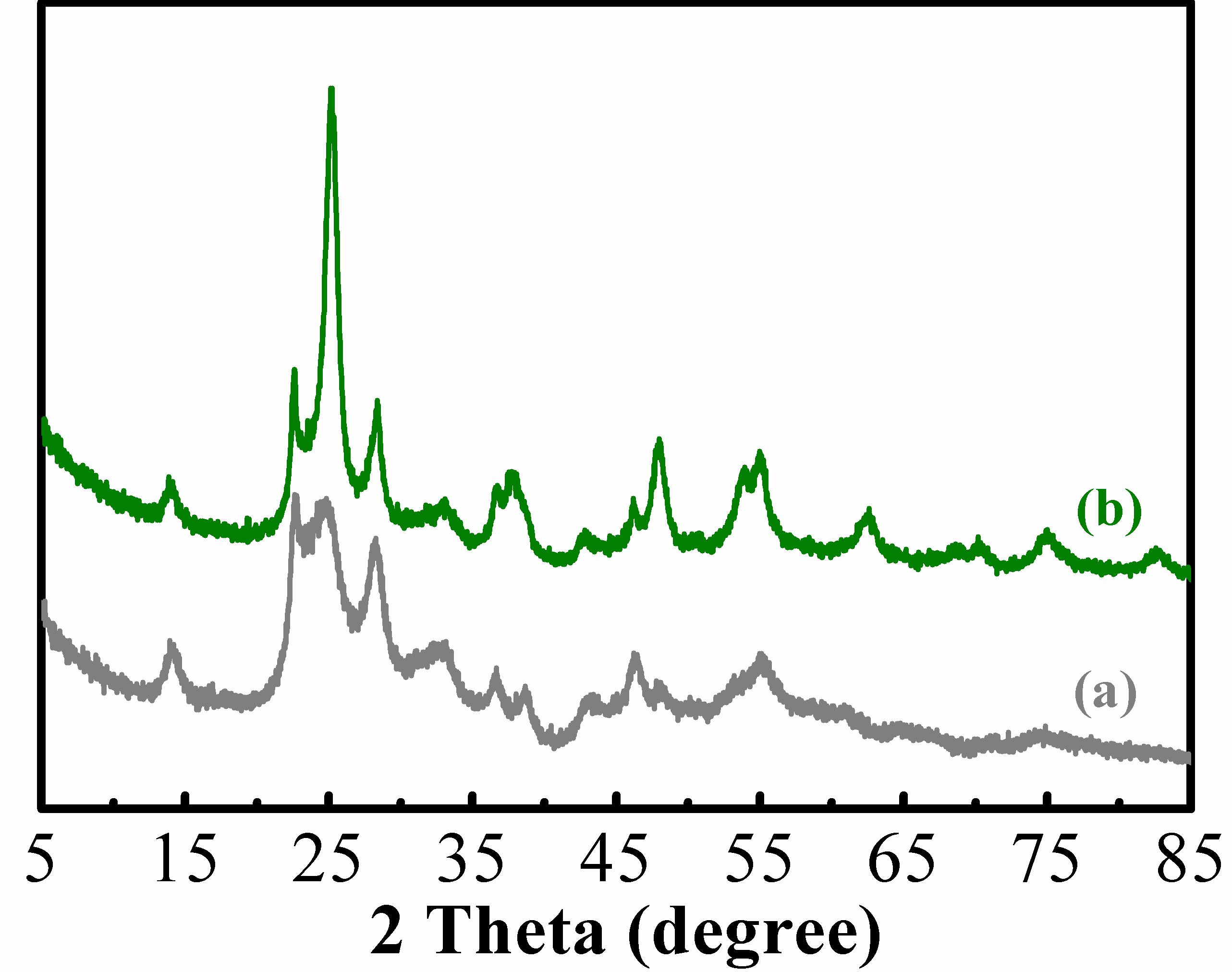


**Figure S11.** XRD patterns of (a) TiO_2_@Nb_2_O_5_-0.1 composites and (b) TiO_2_@Nb_2_O_5_-0.4 composites.


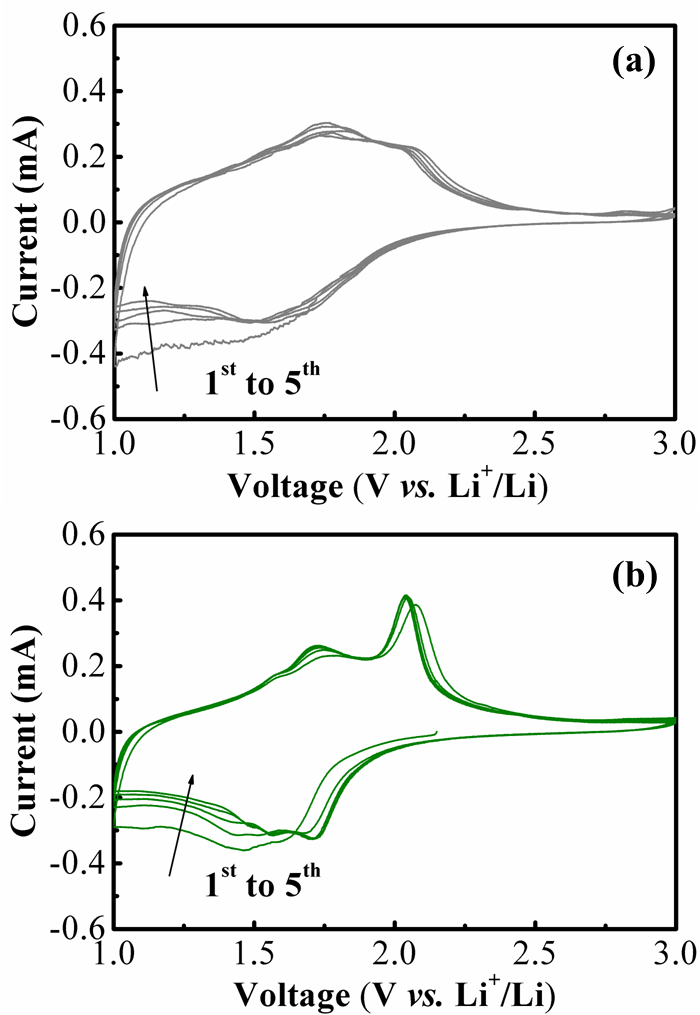


**Figure S12.** CV curves of (a) TiO_2_@Nb_2_O_5_-0.1 composites and (b) TiO_2_@Nb_2_O_5_-0.4 composites at a scan rate of 0.5 mV s^-1^.


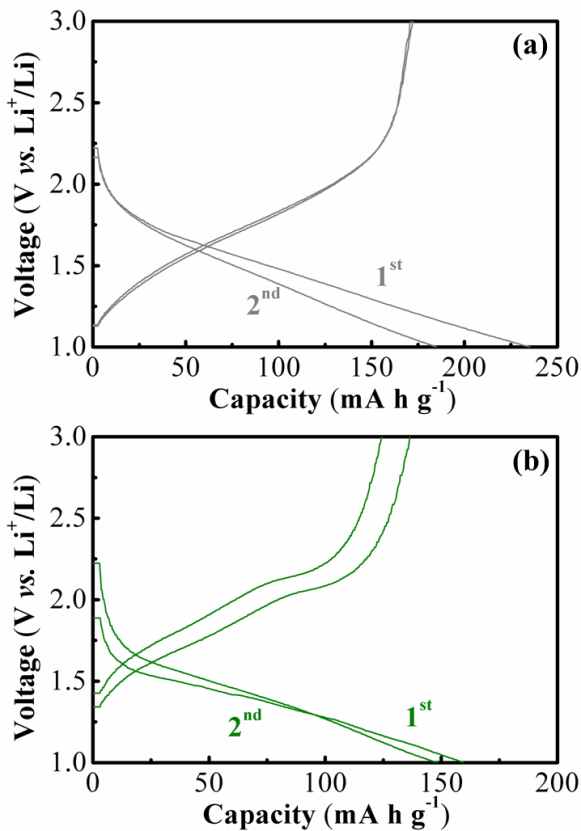


**Figure S13.** Charge-discharge profiles of (a) TiO_2_@Nb_2_O_5_-0.1 composites and (b) TiO_2_@Nb_2_O_5_-0.4 composites at a current density of 1 A g^-1^.


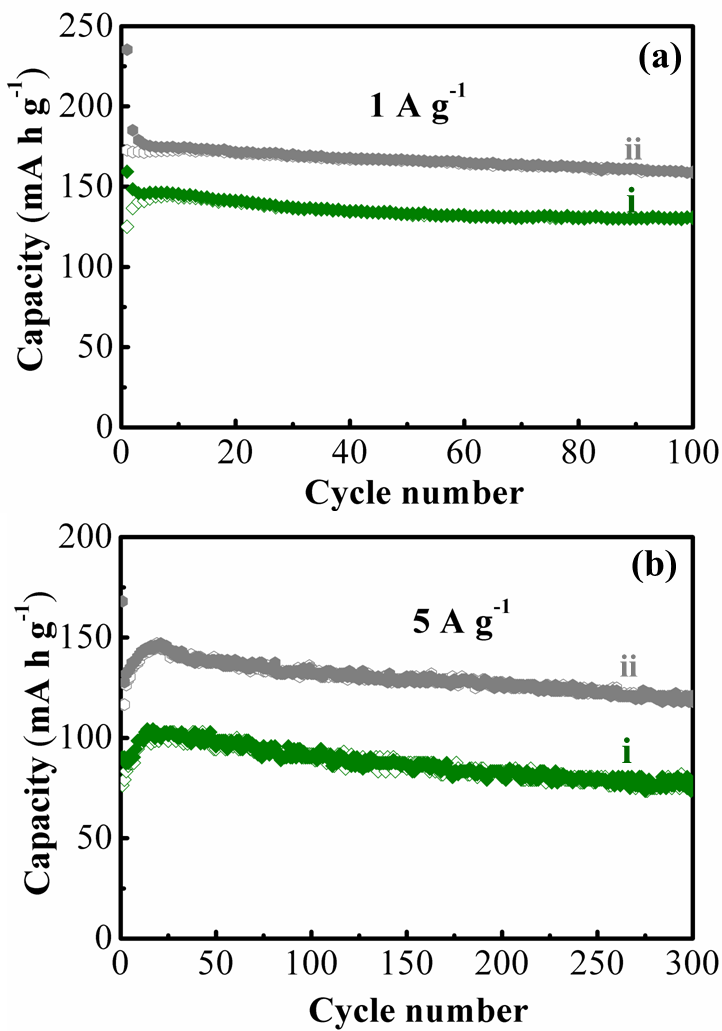


**Figure S14.** Cycling performances of (ii) TiO_2_@Nb_2_O_5_-0.1 composites and (i) TiO_2_@Nb_2_O_5_-0.4 composites at current densities of (a) 1 and (b) 5 A g^-1^.

References

1 Wang, C. et al. Large-scale synthesis of SnO_2_ nanosheets with high lithium storage capacity. *J. Am. Chem. Soc*. **132**, 46-47 (2010).

2 Kodama, R., Terada, Y., Nakai, I., Komaba, S. & Kumagai, N. Electrochemical and in situ XAFS-XRD investigation of Nb_2_O_5_ for rechargeable lithium batteries. *J. Electrochem. Soc.* **153**, A583-A588 (2006).
